# The Future Hospital in Global Health Systems: The Future Hospital as an Entity

**DOI:** 10.1002/hpm.3893

**Published:** 2025-01-02

**Authors:** N. J. Sebire, A. Adams, L. Arpiainen, L. Celi, A. Charlesworth, M. Gorgens, M. Gorsky, F. Magrabi, Y. Nagasawa, C. Onoka, M. McKee

**Affiliations:** ^1^ Digital Research and Informatics Unit NIHR Great Ormond Street Biomedical Research Centre at UCL London UK; ^2^ Department of Social Studies of Medicine / School of Architecture McGill University Montreal Canada; ^3^ Department of Architecture Health and Wellbeing Architecture at Aalto University Aalto Finland; ^4^ Laboratory for Computational Physiology Massachusetts Institute of Technology, and Harvard T.H. Chan School of Public Health Boston Massachusetts USA; ^5^ REAL Centre Team The Health Foundation London UK; ^6^ Digital Health Programme World Bank Group Washington Columbia USA; ^7^ Centre for History in Public Health London School of Hygiene and Tropical Medicine London UK; ^8^ Centre for Health Informatics Australian Institute of Health Innovation Macquarie University Sydney Australia; ^9^ Department of Architecture University of Tokyo Bunkyō Japan; ^10^ College of Medicine University of Nigeria Nsukka Nigeria; ^11^ Department of Health Services Research and Policy London School of Hygiene and Tropical Medicine London UK

**Keywords:** demand, design, future hospital, healthcare system, technology

## Abstract

Health care is changing rapidly. Hospitals are, and will remain, an essential setting to deliver it. We discuss how to maximise the benefits of hospitals in the future in different geographic and health system settings, highlighting a series of cross‐cutting issues. We do this by exploring the evolving roles of hospitals and the main factors that we must consider as they adapt. These include changing population and disease profiles, the impact of evolving technology, and new concepts in hospital design and planning.

Our focus is on delivering high‐quality, patient‐centred care while ensuring equitable access, even if strategic decisions require compromise across these functions. The COVID‐19 pandemic has shown the importance of hospitals in societies while also revealing the limitations of current structures and the potential of technology to transform hospital services within the broader healthcare system.

The aim of this multidisciplinary perspective is to provide an overview of pertinent issues whilst highlighting the challenges and opportunities in optimising future hospital planning, construction, design, and development in high‐income (HIC) and low —and medium‐income country (LMIC) settings.

**Summary**

- The future hospital will have multiple roles and functions, beyond provision of direct patient care, depending on specifics of its social and community setting.
- Changing population demographic profiles will result in increased demand for both primary care/community and hospital‐based healthcare.
- Integrated and multidisciplinary, patient centred care, with increased emphasis on prevention and early intervention will require hospital functionality to support.
- Medical and technology developments will increasingly impact hospital roles and functions, specifically extending their reach beyond the traditional physical confines of the hospital estate.
- Hospital design and architecture can play an important role in role in enabling many aspects of care as well as supporting the health and wellbeing of staff and patients.
- Future hospital development can play an important role in improving access and quality of healthcare in both high and low‐middle income countries, but holistic cross‐domain planning and implementation is required.

## 1 Introduction

Hospitals are central to healthcare systems, combining skills, technology, resources, and knowledge to provide care that cannot be delivered effectively elsewhere. However, hospitals must adapt as population needs and opportunities for health interventions evolve. The future hospital to address the needs of ageing populations with complex health issues, provide increasingly sophisticated care, and incorporate technologies like precision medicine, imaging, and artificial intelligence (AI), while aligning better with developments in community care [1, 2, 3].

Hospitals are not just buildings or facilities but complex systems embedded within the broader healthcare system, with roles in the labour market, community care, research, and regional development, all requiring intersectoral coordination, but limited resources demand difficult trade‐offs. The challenge lies in designing and building hospitals today that can meet future needs while providing the best possible care.

This paper examines the key factors shaping the evolving demands on hospitals and discusses how hospital design and function must adapt to these changes. Topics include building design, technological innovations, and environmental sustainability. We explore how future hospitals can capitalise on opportunities and overcome challenges. However, it is important to note that, while we can offer insights based on current trends and knowledge, predictions remain speculative. By bringing together a multidisciplinary team of authors with extensive experience in these areas, we aim to provide a comprehensive approach to this complex issue.

The original rationale for the hospital was to concentrate scarce resources, including specialist equipment and skilled workforces, to deliver clinical care, teaching, and research. Whilst these principles still apply, changing disease patterns and opportunities to intervene have consequences for staff training and education, while research to determine optimal patient management has become ever more important. The future hospital must focus on treating disease, involving everything from relatively straightforward treatment, such as elective surgery, to complex specialist care, such as precision medicine for cancer. In addition, hospitals should do no harm, and hospital design and processes should minimise iatrogenesis, clinical errors, and hospital‐acquired infections. However it should also actively promote health in therapeutic, healing environments, recognising that this is only possible with an enthusiastic, skilled workforce, demanding measures to attract and retain staff. Finally, it should be recognised that hospitals perform important functions in the wider environment, including as anchor institutions (so‐called because they are rooted in their local community, thereby providing a secure base for development), research and teaching, promoting environmental sustainability and resilience, and interacting in myriad ways with wider healthcare systems in ways that may vary significantly between countries (Appendix A).

To achieve these goals, we need greater co‐design, convening patients, clinicians, health services researchers, and others to engage in design processes in which people are valued and have a continuing voice. Many of the problems experienced by existing hospitals reflect a failure to take account of these considerations (Appendix B). Financial models for such capital developments must be fit for purpose and include features that enable long‐term health system benefits.

## 2 Methods

As a future‐looking perspective that requires thinking outside the box, while this and its accompanying paper draw on the existing literature, especially when looking at history, they go beyond it to consider how the influences on hospitals may change over the next few decades. Our approach is informed by several principles. First, prediction is almost always speculative. Hospitals, like all institutions, are part of complex systems, so their trajectory is characterised by path dependency (many of today's hospitals occupy sites that were selected over a century ago, such as those in London that are adjacent to major railway termini). Changes are non‐linear, exemplified by the s‐shaped curve of technological innovation, are influenced by, and in turn influence many other factors. For example, changes in technology have changed clinical pathways and led to the emergence (and disappearance) of clinical roles. Hence, a traditional systematic review would not have been appropriate. Rather, we selected a panel of authors with expertise in a range of areas relevant to the hospital. These include clinical medicine, architectural and public health history, architecture and wellbeing and urban design, data science and artificial intelligence, economics, and health systems. Each contributed insights into likely futures for the hospital that were integrated into a narrative text.

## 3 Brief Historical Perspective of Hospital Development

The hospital originated as a religious refuge for the sick, providing shelter, food, and warmth, but not medicine [4, 5, 6]. In mediaeval Europe, such institutions included alms houses, leper houses, and refuges for the poor. Renaissance Italy expanded the role of hospitals to treat the sick, the destitute, and unsupported mothers [7]. Therapeutic hospitals for the acutely ill poor emerged later, often funded by charity or aristocratic endowment. By the 19th century, teaching hospitals in cities like Paris and Edinburgh advanced scientific medicine through clinical observation and novel technology. By the 1930s, hospitals evolved to treat acute illness using tools like stethoscopes, x‐rays, and anaesthesia. Today, hospitals vary widely, encompassing teaching and non‐teaching, general and speciality, somatic and psychiatric, acute and chronic, as well as medical and social care.

In 19th‐century Europe, wealthier patients began paying daily charges, and mutual funds grew after Bismarck introduced social health insurance in 1883 [8]. In the USA, private hospitals initially relied on direct billing but shifted to private and voluntary insurance in the 1930s. Public funding for older and low‐income patients followed, with Medicare and Medicaid in the 1960s [9, 10]. From the 1980s, the American model of hospital user fees influenced global health, promoted by the World Bank [11]. While these fees supported weak health systems, they also created access barriers, fostering inequities and welfare losses [12].

## 4 Hospitals and Population Need

An optimal health system aligns hospital capacity with demand, influenced by demographics, disease burden, and primary care availability. Over time, the focus has shifted from communicable diseases to conditions like cancer and cardiovascular conditions. Acute care beds in OECD countries have declined due to advances like day surgery, intravenous anaesthetics requiring shorter recovery, and expedited discharge [13]. However, hospitals may still be misaligned with population needs, leading to geographical inequities [14]. While resource allocation based on population needs can help, political challenges remain.

## 5 Drivers of Future Hospital Requirements

Four key factors will shape future hospital demands. First, changing care needs are driven by ageing populations, varied life experiences, and evolving risk factors. Rates of conditions like smoking‐related diseases have declined, but chronic conditions and age‐related illnesses are rising. Older adults, though healthier, live with more chronic issues, impacting admissions and care complexity, while advances like precision medicine offer new interventions. By 2050, the global population aged 80+ will triple to 425 million, with two‐thirds in LMICs.

Complex morbidities, including multiple chronic conditions and cancer, increasingly require specialised staff and technology [15, 16, 17, 18]. Chronic musculoskeletal and mental health disorders also contribute to rising demand [19], with UK hospital care needs projected to grow 40% in coming decades, especially for elderly, outpatient, and elective care [20]. Over 20% of emergency visits in Europe already involve older adults, often due to a lack of alternatives [21]. Social and economic factors tied to age predict higher hospital use [22], despite expanded community care, with rising demand for day‐care services and step‐down units [23].

An ageing population affects healthcare not only by increasing patient needs but also by shrinking the available health workforce. Japan, with its ageing population and low immigration, faces severe staff shortages, driving the adoption of technological innovations [24]. Globally, migration exacerbates healthcare worker shortages in LMICs.

Future funding models may shift from activity‐based billing to value‐based payments or population health approaches, integrating hospitals into broader care systems and emphasising health promotion and disease prevention [25]. In HICs, payment systems increasingly view hospital care as part of a care pathway, though coordination between separate providers remains a challenge. Economies of scale may reduce costs, but risk affects quality, staffing, and accessibility, and these approaches raise challenges where hospitals and other healthcare providers are separate organisations [26].

Technological advances, defined as the ‘application of organised knowledge and skills in the form of devices, medicines, vaccines, procedures, and systems developed to solve a health problem and improve quality of lives’ [27], are transforming hospital design and services. These innovations enable community‐based care, early intervention, and reduced reliance on traditional hospital settings. For example, Estonia's healthcare digitalisation has shifted focus from hospitals to community care, decreasing hospital numbers [28]. Specialist centres may now serve populations across borders, altering care delivery, reimbursement models, and user preferences, a trend growing in both HICs and LMICs.

Advances in AI and machine learning will impact care pathways [29]. The less contentious areas relate to straightforward tasks such as interpreting some medical images (e.g., retinal photographs) and assisting with documentation [30]. Some see generative AI as transformative but, so far, most applications have been limited. It also carries risks like accountability, bias in algorithms, privacy concerns, and potential for misinformation [31]. Clinicians will have to have sufficient trust to use these tools but not so much that they allow them to override their judgement, something that explainable AI (XAI) aims to address [22, 32, 33].

Hospitals also have a global impact. As major contributors to carbon emissions, hospitals must adopt eco‐friendly practices across supply chains, infrastructure, energy use, transport, and care models [34].

## 6 Definitions and Functions of the Future Hospital

Hospitals are defined by their provision of continuous specialist inpatient care, though their roles extend to teaching, training, research, and community engagement. They may specialise in emergency care, maternity, or paediatrics, with some facilities arising as a consequence of historical circumstances, like tuberculosis sanitoria [35]. As healthcare focuses more on overall well‐being, patient‐centric care will increasingly replace traditional, clinician‐focused models, supported by digital access to health information and remote care options [36].

Future hospitals will serve as hubs of skilled healthcare staff and advanced resources, though this will vary according to availability of resources. In some LMICs, hospitals may be the only sites with constant physician availability, while hybrid models combining onsite and remote care are growing, accelerated by the COVID‐19 pandemic.

Traditional definitions generally fail to adequately represent adequately evolving ‘hybrid hospital models’, delivering both onsite and remote care. These models have developed in response to demographic and epidemiological trends and the availability of technology, which accelerated during the COVID‐19 pandemic. Indeed, ‘hospitals without walls’, based on teleconsultations and remote monitoring, have been established, and there are examples of ‘hospitals’ with no inpatient beds onsite (although this raises terminological issues) [37, 38].

In addition to direct clinical care, future hospitals must support elements of other activities across the healthcare system, including training healthcare professionals and aspects of patient education and/or preventative care, although such activities are often inconsistent with current remuneration models [39, 40].

Medical research ranges from laboratory studies to clinical trials and is concentrated in teaching hospitals and medical schools that enable access to patients and governance structures. This disproportionately favouring academic centres in HICs. Future hospitals should do their bit to ensure that participants in research reflect their populations, actively supporting research by, for example, recruiting patients to trials and evaluating new models of care [41].

Future hospitals will continue as specialist technology hubs, providing services like diagnostic imaging, laboratory medicine, and emerging technologies, often through community referrals [2]. In the UK, 70% of clinical decisions rely on hospital laboratory tests, regardless of care setting [42]. Hospitals also house costly facilities like operating rooms and ICUs, with demand increasing. Currently, nearly 10 million surgical procedures are performed annually in UK hospitals for a population of 65 million [43].

For all these reasons, most conceptual healthcare models view hospitals as central to healthcare systems [44] but they also play a vital role in local communities. They drive local economies as major employers, often promoting gender equality and good working conditions and providing facilities like public spaces. Hospital grounds may function like public parks, and in some LMIC settings, the hospital may be one of the only organisations with consistent supplies of clean water and electricity [45], whilst also serving as a place of safety in conflicts.

The future hospital must meet these evolving demands while emphasising patient‐centred care in a supportive setting, including awareness of impacts on local ecosystems and the environment. Hospitals must deliver increasingly advanced and complex care, such as precision medicine, cancer treatments, and immunotherapy, while supporting other activities in the community and through remote diagnostics and consultations.

## 7 Future Hospital Design

Hospitals have traditionally been places of refuge, rehabilitation, and specialised medical care, optimised for medical technology and functions [46]. The concept of the hospital has evolved from simply providing specialist services to focussing on patient care, with greater emphasis on the quality of the hospital environment, appealing design, and the inclusion of green spaces as ‘places for healing.’ Hospital design can also support sustainability and create more inviting spaces, such as incorporating retail stores within hospital sites [47]. These features promote calm, improve patient and staff experiences, and support functions like infection control and nursing efficiency, all while maintaining the core role of treating the seriously ill [48]. For example, emergency departments, operating rooms, and intensive care units remain essential, along with services like diagnostic imaging and laboratories. However, some functions traditionally done in hospitals, such as outpatient care or virtual services, may shift elsewhere. Hospitals may also increasingly serve broader community functions, like art exhibits, food courts, shopping areas, and social programs, helping to improve public health and reduce the need for hospital care [49].

Hospitals have traditionally been seen as ‘process buildings’ for medical care, but they are also places where patients and families experience emotionally intense events and where staff often spend much of their working lives. As a result, the built environment must be carefully planned to promote healing and support staff retention. The needs of specific patient groups are also increasingly recognised, such as separate areas in emergency departments for those with mental health issues, or specialised spaces for obstetric and paediatric care. Following the COVID‐19 pandemic features like proper ventilation have become essential [50]. These evolving requirements, while ensuring the ability to respond to emergencies and epidemics, demand greater flexibility in hospital design. However, incorporating these features can increase construction complexity and costs, especially for systems like air conditioning, electrical fittings, automated transportation, and information architecture, which may have different lifespans than the building or medical equipment.

Hospitals are often located in densely populated cities on small, expensive plots, so space must be used efficiently. They typically employ compact, multistorey designs that depend on artificial ventilation and heating environments, raising operating and maintenance costs [51]. Since hospitals run continuously, mechanical components often have shorter lifespans and require regular servicing. Additionally, hospitals need system redundancies to handle power failures or other emergencies. For instance, equipment may require uninterruptible power supplies and generators, especially to manage surges in demand, such as the oxygen shortages experienced in some LMIC hospitals during the COVID‐19 pandemic.

Hospitals are often composed of multiple buildings, like a small town, each serving different functions and having specific technical requirements, such as weight restrictions for shielding MRI and other imaging equipment. Hospital designs should prioritise reducing long‐term operational costs over focusing solely on capital expenses, considering factors like environmental impact and sustainability. However, creating high‐quality and suitable facilities in LMICs presents challenges, as it may increase both construction and operational costs while still adhering to these design principles.

## 8 Data and Digital Technology in Future Hospitals

The impact of digital services, data, and technology will have a major influence on future hospital function and design. [52]^,^ Whilst affecting most aspects of hospital care to some degree, the greatest impact may be on care that is not restricted ‐geographically (virtual appointments/telemedicine, virtual‐wards), home monitoring for early detection of disease and ongoing care, digital platforms for staff‐patient interactions, as well as robotics and smart buildings within the hospital. Large hospitals will increasingly become data science/AI‐enabled organisations, generating and leveraging huge volumes of data from electronic health records (EHR), imaging, diagnostics and sensors on patients beyond their walls (Appendix C).

However, there is still great uncertainty about how such developments should influence hospital planning and design and the actions required to achieve any benefits. What is certain is that they will require investment in equipment and infrastructure, staff and patient upskilling, and changes in practice. These will require expertise in procurement, emphasising the involvement of practitioners and patients [53]. Whilst technology developments may be rapid, creating the systems and skills to implement them will be more difficult; for example, home monitoring requires minimal new hardware but significant changes to clinical pathways.

Whilst technology‐enabled healthcare services will increase, their scale of adoption will likely differ across organisations and countries, with a potential divide in digital access. The COVID‐19 pandemic stimulated the use of telehealth, such as virtual consultations [54]. and remote monitoring, making patients and staff more comfortable with ‘smart environments’ but ensuring equity of access remains challenging [55, 56]. The WHO digital health strategy emphasises that digital tools should be integrated into future healthcare provision, ensuring they are ethical, safe, secure, reliable, equitable and sustainable to support equitable, universal access to health services whilst enhancing efficiency and sustainability [57]. Whilst opportunities to achieve positive impacts of health technology are manifold, implementation of digital technology in LMIC settings faces challenges, such as requirements for appropriate physical environments, resources and infrastructure, staff and patient/user education, human capacity, financial investment, data connectivity, as well as issues related to legacy infrastructure, ownership, privacy, security, and implementing standards and technology flow. Furthermore, AI/digital technologies in HICs and LMICs will contribute to climate change because of their enormous energy requirements, although this could be offset by benefits such as reducing patient travel to the hospital [58].

During the COVID‐19 pandemic, ‘virtual hospitals’, with remote consultations and/or virtual wards, expanded greatly [59]. Hospital at‐home programmes for older people using telemedicine achieved improved outcomes, reduced in‐hospital stays, and cost savings compared to traditional inpatient care [60]. However, success is not guaranteed, and implementation requires environments to consult with appropriate technology [61]. Whilst potentially mitigating future capacity and staffing issues, the real‐world effectiveness of virtual wards remains to be determined across care settings. In the United Kingdom during the COVID‐19 pandemic, COVID virtual wards did not significantly reduce readmissions, intensive‐care admissions, or deaths [59]. Nevertheless, aspects of traditional hospital‐based care will likely become less dependent on delivery locations, allowing flexibility in care models and reducing real‐estate requirements for some hospital services.

The application of hospital digital technology has potential benefits and unintended consequences [62, 63]. Principles for the successful adoption of technological solutions include: digital technologies should be problem‐based rather than technology‐driven; digital technology should be a means to address priority issues rather than a separate goal; and digital technologies should be considered in the context of overall service and hospital operations, to ensure that downstream effects are recognised and benefits can be realised. Finally, technology solutions only yield benefits when appropriately implemented, and local sociotechnical factors should be considered [64].

Digital health implementation is complex, characterised by tensions and trade‐offs by various stakeholder groups with conflicting interests. For example, a review of the implementation of the NHS Care Record Service (covering 50 million English residents) [52] identified 15 interlinked organisational, human and technological factors required for success [65]. Similarly, medication management systems are well‐studied, resulting in reduced medication errors, but there is limited evidence regarding adverse drug events, mortality, or other outcomes. Additionally, medication alerts cause ‘alert fatigue,’ with 50%–96% being overridden in routine clinical practice and the alert burden increasing by a factor of six [66]. Similarly, video consultations may be beneficial, but embedding them in routine practice can be challenging [67].

Beyond technology and infrastructure, hospital staff, such as IT personnel, clinical staff, informaticians, and patients, will have varying information and communication needs and skills. This is important since patient harm associated with digital health and AI tools is more often due to human factors than technical issues [68, 69].

External rules and regulations will guide the implementation of digital health technologies, ensuring they are ethical, safe, sustainable, and compliant with privacy and security requirements. Tools must be monitored for benefit and harm, involving co‐development with patients and staff and considering the entire workflow and lifecycle. Future hospitals may need clinical informatics/AI units to coordinate these activities [70].

Digital technologies offer a novel approach to addressing social determinants of health and providing services to those excluded by traditional methods, such as through telemedicine and digital symptom checkers. These technologies potentially democratise health information. However, this should not replace addressing other structural drivers of health [71]. In some LMICs, populations may have greater internet access than expected due to affordable smartphones and high digital literacy, despite impoverished conditions. In contrast, many in HICs (up to 25% of Americans) lack broadband internet, even with access to education, health, and social services. This creates a risk of ‘digital divides’ related to network access and digital literacy.

Certain digital tools may negatively impact specific groups due to limited ethnic and geographic diversity in healthcare data, leading to biases and health inequalities. For example, medical devices like pulse oximeters are less accurate on darker skin tones [72]. While digital health tools and AI could transform healthcare in LMICs, which face resource shortages and medical workforce gaps, their effectiveness depends on appropriate data. Disparities in data infrastructure investment between LMICs and HICs may worsen data inequity, leaving countries with less health‐specific data less likely to benefit and potentially exacerbating health inequalities [73].

Data availability for digital health tools, including algorithms, requires skilled healthcare providers and non‐clinical team members. Investments in team learning and co‐development are essential for scaling data systems beyond single organisations. Advanced hardware and software can only generate population‐wide benefits if the organisational culture fosters collaboration and innovation and if the public has confidence in data security and safety.

## 9 The Particular Challenges Facing LMICs

While we have set out a vision for the future hospital, we need to face the reality that many LMICs will face major challenges in realising it. Limited financial resources restrict investments in cutting‐edge infrastructure, as high upfront costs for eco‐friendly materials and advanced technologies often outweigh budgetary capacities. Sustaining operations also requires significant, ongoing expenditure.

Technological and infrastructure deficits exacerbate these issues. Advanced designs rely on robust digital ecosystems, such as electronic health records and AI‐driven tools, which are often unavailable due to unreliable internet, power supplies, and technical expertise. Retrofitting legacy infrastructure to accommodate new technologies is complex and costly.

Human resource shortages further limit implementation. Many LMICs lack skilled architects, engineers, and healthcare workers trained to manage advanced hospital systems. Brain drain compounds these issues, reducing the local talent pool.

Governance and policy deficiencies also play a critical role. Weak regulatory frameworks, corruption [74], and bureaucratic inefficiencies delay hospital projects and deter international investment. Cultural and contextual factors present additional challenges. Advanced hospital models, often designed for high‐income settings, may not align with the community‐centric healthcare delivery systems in LMICs. Hospitals frequently serve as community hubs, providing essential utilities like water and electricity during crises, functions that specialised, technology‐driven designs may neglect.

Environmental considerations further complicate adoption. Many LMICs are highly vulnerable to climate change, yet sustainable hospital designs often require costly energy‐efficient technologies. Implementing such systems becomes even more challenging in regions with unreliable power supplies.

Overcoming these barriers requires multi‐pronged strategies, including international collaboration, adaptive designs tailored to local contexts, and innovative financing like public‐private partnerships. Capacity building, equitable technology transfer, and governance reforms are essential. Incremental advancements, rather than wholesale adoption of high‐tech systems, can bridge gaps while ensuring hospitals address both healthcare and community needs.

## 10 Conclusions

Multiple factors drive the need for structured planning of future hospital provision, including sociodemographic changes, technological advancements, shifting disease profiles (ageing, multimorbidity, mental health), advances in clinical care, health service design, staffing, and changing work environments through technology. Future hospitals should support well‐being and health, with smart buildings optimising technology use, impacting care pathways, remote monitoring, telemedicine, outpatient care, AI‐based diagnostics, and integration with urban communities. However, uncertainty remains regarding the pace and extent of these changes, including the long‐term impacts of diseases like COVID‐19.

While hospital functions in HICs will evolve, LMICs face greater challenges due to fewer resources, lower healthcare spending, rising life expectancy, and increasing chronic diseases. Addressing these issues offers opportunities to improve system costs, patient safety, access, experience, staff retention, outcomes, economic impacts, sustainability, equity, and public health.

## Author Contributions

N.J.S., M.M. conceived the series and wrote the initial manuscript draft. All authors contributed to working groups, the development of specialist content and contributed to the writing of the final manuscript for submission.

## Ethics Statement

Perspective hence ethical approval not required.

## Conflicts of Interest

The authors declare no conflicts of interest.

## Data Availability

The data that support the findings of this study are available on request from the corresponding author. The data are not publicly available due to privacy or ethical restrictions.

## Appendix A Major Architectural Considerations for Modern Hospital Design

### 1. Healing Environments

The hospital is more than a ‘process building’ for medical diagnostics and treatment but should be a structure with a role in healing. Proponents of Evidence Based Design (EBD) have mapped qualitative elements that contribute to holistic healing; daylight and exterior views, warm colours and materials, biophilic elements, soft surfaces for acoustics, access to the outdoor areas, and dedicated spaces for families are now standard features in healthcare design. We expect this big‐picture approach to continue.

### 2. User‐Centred Design

Hospitals are where staff, patients and families experience intense events, and the concepts of ‘patient experience’ and ‘patient and family centred care’ are now foundational in planning. Hospitals are also workplaces and therefore staff wellbeing is a growing design consideration in an age of staff shortages. Both patients and staff should participate in hospital design. We suggest users are fully engaged in the design of future hospitals.

### 3. Changes in Disease Profiles

Disease profiles are changing, and patients present with more multiple comorbidities. The growing needs of mental health and substance use emergencies and hospitalisations merit separate consideration, such as emergency departments with spaces for mental health. Some hospitals have entirely separate mental health emergency departments with attached short stay units. These changing profiles mean that architects must adapt in their design decisions.

### 4. Changes in Demographics

Ageing populations result in more people with complex, simultaneous, and overlapping health problems. Dementia, hearing and vision loss, and mobility issues change our perceptions and experiences of architecture. Sometimes these conditions require special buildings for care that are related to but different than hospitals. Designers of the future should re‐focus and improve longterm residential care, chronic care, and end‐of‐life care facilities, freeing up space in acute‐care hospitals.

### 5. Changes in Technology

Technologies are constantly changing to advance care and change care delivery, such as remote monitoring with smaller and portable sensors. Artificial intelligence will impact administration, diagnostics, triage, and clinical care, including the potential to flag irregularities in processes and outcomes. Consequently, future hospitals will require more ‘non‐human’ spaces. Since such rooms can be windowless, future hospitals may have deeper footprints or include underground facilities.

### 6. Changes in Service Delivery

Many care needs can be delivered outside the hospital. Increasing rates of same‐day and ambulatory services have resulted in ‘hospitals with no beds.’ Many hospitals focus on one service, such as ophthalmology or joint replacement. Centres of excellence and large trauma centres are typically in cities, while community hospitals offer generalist care in less densely populated areas. Future hospitals are likely to continue to increase integration with surrounding buildings.

### 7. Climate Effects

Hospitals continue to be energy intensive buildings that operate continuously and use 2–3 times the amount of energy of other commercial buildings. Future hospitals will likely have sophisticated building management systems that optimise energy use, as well as built‐in climate mitigating features such as green roofs, permeable fenestration, and super‐efficient envelopes. Hospital waste will likely be highly scrutinised. Support for the paperless hospital will spread globally.

### 8. Renovations

Inspired by the global embrace of recycling, the reuse of buildings is a popular trend. We expect future hospital needs to be satisfied or adapted within existing structures, when available, avoiding unnecessary demolition and the high cost of new buildings. Hospitals will be designed and built so that flexibility, adaptability and standard solutions enable a maximum level of future‐proofing. We also expect that fewer cars will be accommodated on hospital sites, freeing up land for expansion projects to existing buildings.

### 9. Crisis Resilience

The prevalence of global and local crises continues to rise and Hospitals must respond to political, military, climate‐related, public health and/or environmental disasters. In case of a power outage, a hospital needs to operate independently, requiring back‐up power for clinical equipment and data systems. Bed counts may need to expand for surge capacity and certain areas of the hospital may need to be able to be isolated with infectious diseases. Hospitals in risk areas need evacuation plans. We expect the role of crisis planning to rise in the future hospital.

### 10. Health Equity

Access to quality healthcare varies widely both in the industrialised world and LMICs. Support of LMICs and development of their healthcare infrastructure is imperative. HICs must invest in collaboration with populations in need, including those facing displacement. Portable, relocatable, and mobile hospitals and clinics will continue as a significant future trend.

## Appendix B Major Reasons for Failures of Previous Hospital Building Projects

### Obsolescence

Hospital architecture can never adequately accommodate the ever‐changing needs of modern medicine. The consequence of this failure is continuous renovation, resulting in hospitals comprised of layers, each representing a particular era. Sometimes, hospital planners declare these patchwork structures obsolete, claiming that no further modernisation is possible. Obsolescence in hospital design can be something as basic as rooms or corridors which no longer support certain equipment.

### Changes in Care Delivery

The last two centuries have seen massive changes in care delivery. Today's best practices, in some cases, are the opposite of what was prescribed in the past and in the most extreme examples, older buildings might even cause harm. For example, 19th‐century psychiatric hospitals accommodated patients at the edges of cities, with little connection to neighbourhoods or services. Today's mental health facilities, by contrast, are often community‐based and/or integrated with other services.

### Demographic Changes

Demographic changes and rapid urbanisation can pose a challenge to hospitals that may originally have been established in thriving locations. Remote areas with declining populations may see their community hospitals declared inefficient and eventually closed. Additionally, the ageing population may mean we need more residential care and supported living in the future. These changes underscore the need for careful demographic analysis and demand projections in hospital planning, even though some may be unpredictable.

### Politics and Policies

Hospitals are expensive and essential buildings. Apart from keeping a population healthy, hospitals are also major employers. A hospital closure can be devastating for a town or city. The location, expansion, and maintenance of hospitals are therefore important for political purposes. Occasionally, hospitals that could be updated are declared unusable for financial or political gain.

## Appendix C Artificial Intelligence, Digital Determinants of Health and the Future Hospital

Digital Determinants of Health are factors intrinsic to technology that when applied to the provision of healthcare services can have a major impact on health outcomes [57]. These include aspects such as ease of use, usefulness, interactivity, digital literacy, accessibility, affordability, algorithmic bias, technology personalisation, data poverty, and information asymmetry. In 2020, digital health entered a new era with the mass adoption of telemedicine due to the COVID‐19 pandemic. Further, applications for Artificial Intelligence (AI) and machine‐based learning in healthcare are expanding rapidly [75, 76, 77].

There is a diverse range of work carried out confirming the effectiveness of digital technology in developed countries, but these studies also characterise a variation in the demographic reach even in HICs, with the lower socio‐economic cohorts inevitably left behind. The digitally excluded include the elderly, the disabled, including those who are visually‐ and hearing‐impaired compared to the wider population [78]. Differences in rural versus urban infrastructure limit access to digital systems [79, 80], but, notably, engagement is not solely reliant on availability, as those with lower income demonstrate reduced engagement even where such systems are available [81]. Differential experience with digital health tools has substantial population health implications health systems globally. Whilst technology has the potential to democratise and decentralise healthcare knowledge and access, it may also intensify disparities by further widening the divide between those who are privileged and those who are marginalised. These emerging drivers of health disparity call for purposeful, equity‐focused strategies to ensure that technological innovation benefits all without exacerbating disparities [82].

Additionally, since 2022 there have been groundbreaking advances in the field of AI with the release of image‐generating technologies (such as latent diffusion and stable diffusion), and several large language models (LLMs) associated chatbots. Fundamentally, LLMs make probabilistic suggestions regarding text that belongs together in a sequence, based on statistical models trained on billions of examples of text ingested from various sources, some including medical documents and textbooks. At the clinical level, such models have demonstrated the ability to answer complex, context‐specific medical knowledge questions accurately [83, 84, 85], as well as to structure and summarise clinical data [86]. It is therefore likely that in future hospitals, many aspects of day‐to‐day clinical practice may be LLM‐facilitated. Models may, for example, be able to generate brief summaries of a patient's past medical history, identifying important but easily missed facts within hundreds of pages of past notes. They may automatically scribe notes from clinical interactions, helping to address the significant paperwork burden faced by providers like generating letters in response to insurance denials [87]. LLMs may offer nuanced, context‐specific answers to medical questions, such as generating differential diagnoses for a complicated set of symptoms, or offering “second opinion” management suggestions based on recent clinical guidelines [88].

LLMs are very powerful tools in sifting through content beyond the capabilities of experts, or even groups of experts, and extracting knowledge. However, the issue of data bias must be addressed before LLMs and other artificial intelligence (AI) technologies can be leveraged for maximum global value. The body of knowledge that LLMs train on, both medical and beyond, is dominated by content and research from well‐funded institutions in high‐income countries. It is not representative of most of the world. Only a handful of hospitals and health systems have data pipelines and expertise to harness the promise of AI. Without a roadmap to build these technologies with an equity‐focused design, AI is likely to contribute if not magnify existing health disparities. Furthermore, in addition to bias from sampling selection of the training population, some technologies may perform differently across patient subgroups (e.g. pulse oximetry and wearable sensors), and AI models may learn ‘hidden’ sensitive attributes that should not affect decision making but which are embedded in the dataset, such that existing outcome disparities become encoded into the algorithms. Such factors represent major challenges for developing scalable AI tools using real‐world data.
